# A Smartphone App to Foster Power in the Everyday Management of Living With Schizophrenia: Qualitative Analysis of Young Adults’ Perspectives

**DOI:** 10.2196/10157

**Published:** 2018-10-01

**Authors:** Malene Terp, Rikke Jørgensen, Birgitte Schantz Laursen, Jan Mainz, Charlotte D Bjørnes

**Affiliations:** 1 Department of Psychiatry Aalborg University Hospital Aalborg Denmark; 2 Department of Clinical Medicine Aalborg University Aalborg Denmark; 3 Clinical Nursing Research Unit Aalborg University Hospital Aalborg Denmark; 4 Department for Community Mental Health Haifa University Haifa Israel

**Keywords:** mental health, mHealth, mobile app, participatory design, patient empowerment, patient involvement, patient participation, schizophrenia, smartphone, young adults

## Abstract

**Background:**

Literature indicates that using smartphone technology is a feasible way of empowering young adults recently diagnosed with schizophrenia to manage everyday living with their illness. The perspective of young adults on this matter, however, is unexplored.

**Objective:**

This study aimed at exploring how young adults recently diagnosed with schizophrenia used and perceived a smartphone app (MindFrame) as a tool to foster power in the everyday management of living with their illness.

**Methods:**

Using participatory design thinking and methods, MindFrame was iteratively developed. MindFrame consists of a smartphone app that allows young adults to access resources to aid their self-management. The app is affiliated with a website to support collaboration with their health care providers (HCPs). From January to December 2016, community-dwelling young adults with a recent diagnosis of schizophrenia were invited to use MindFrame as part of their care. They customized the resources while assessing their health on a daily basis. Then, they were invited to evaluate the use and provide their perspective on the app. The evaluation was qualitative, and data were generated from in-depth interviews. Data were analyzed using a hermeneutical approach.

**Results:**

A total of 98 individuals were eligible for the study (mean age 24.8, range 18-36). Of these, 27 used MindFrame and 13 participated in the evaluation. The analysis showed that to the young adults, MindFrame served to foster power in their everyday management of living with schizophrenia. When MindFrame was used with the HCPs consistently for more than a month, it could provide them with the power to keep up their medication, to keep a step ahead of their illness, and to get appropriate help based on their needs. This empowered them to stay on track with their illness, thus in control of it. It was also reported that MindFrame could fuel the fear of restraint and illness exacerbation, thereby disempowering some from feeling certain and secure.

**Conclusions:**

The findings demonstrate that young adults diagnosed with schizophrenia are amenable to use a smartphone app to monitor their health, manage their medication, and stay alert of the early signs of illness exacerbation. This may empower them to stay on track with their illness, thus in control of it. This indicates the potential of smartphone-based care being capable of aiding this specific population to more confidently manage their new life situation. The potentially disempowering aspect of MindFrame accentuates a need for further research to understand the best uptake and the limitations of smartphone-based schizophrenia care of young adults.

## Introduction

### Background

It is well established that self-management knowledge and skills are the cornerstones of preventing exacerbations and relapse of psychotic illness [1-4]. However, many young adults recently diagnosed with schizophrenia skip their clinical visits [5,6], leaving them with only little knowledge and skill power to manage everyday living with the illness efficiently. This causes a serious threat to their current and future health and quality of life [7,8]. This proves the need to find new and innovative approaches to build competencies to empower them to manage the illness in the context of their daily lives. An approach could be smartphone-based care. The pervasive nature of the smartphone and smartphone apps allows to monitor health and for customized information and self-management tools to be disseminated in real time and in real-life settings [1,9-14], where and when it is needed [15-16].

### Smartphone Apps for Schizophrenia Care

Smartphone apps have been developed for schizophrenia care [17-25], yet only limited attention has been paid to mobile health (mHealth) apps to provide illness management support to individuals with schizophrenia outside the confines of the mental health clinic [26]. A review of smartphone apps for schizophrenia identified only 1 app providing this kind of support [27,28]. This app offered prescheduled and on-demand resources to facilitate symptom management, mood regulation, medication adherence, social functioning, and improved sleep. Evaluation of the app in 33 individuals with schizophrenia or schizoaffective disorder showed that the participants were willing and capable of using the app independently in their own environment [28].

Although sparse, the existing literature indicates that a smartphone app is a promising way to empower young adults recently diagnosed with schizophrenia to manage everyday living with their illness. The viewpoint of this matter from the perspective of those living with the illness as part of their daily lives, however, is unknown. As interests in smartphone apps in schizophrenia care grow [14,17,20,24,25,29], this seems increasingly important to explore.

Qualitative research is a systematic inquiry seeking to explore, and eventually understand, the experiences of a particular group of people [30,31]. A qualitative inquiry may provide insider perspectives to aid the understanding of the viability of apps to make those recently diagnosed with schizophrenia more capable and confident in managing their lives. Using a qualitative inquiry, the objective of this study was, therefore, to explore how young adults recently diagnosed with schizophrenia used and perceived a smartphone app (MindFrame) as a tool to foster power in the everyday management of living with their illness.

## Methods

### MindFrame

**Figure 1:**
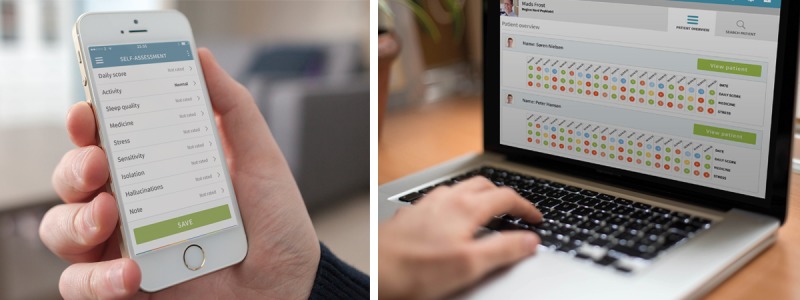
Screenshots of the MindFrame app and the affiliated website.

Using participatory design thinking [32-35] and methods [36-38], MindFrame was iteratively developed to run on the Monsenso mHealth platform powered by Monsenso ApS. The platform has been technically and clinically validated in various clinical evaluation studies and randomized clinical trials (RCTs) [39]. First, interviews were conducted with young adults recently diagnosed with schizophrenia to explore their perspective of needs to be supported in the everyday management of living with the illness and to generate ideas of using the smartphone to accommodate the needs [40]. Then, young adults recently diagnosed with schizophrenia, health care providers (HCPs), a researcher, and software designers collaboratively designed resources to accommodate the needs [41]. Figure 1 shows MindFrame, which consists of a smartphone app that allows young adults diagnosed with schizophrenia to access resources to aid their self-management. The app is affiliated with a website to support collaboration with their HCPs. A comprehensive description of the resources in MindFrame, including its aims, capabilities, and intended use, is provided in Table 1.

**Table 1:** MindFrame app resources.

Resource	Aim of resource	Capabilities of resource	Intended use of resource
Self-assessment	Monitor health	Data input to report the mental health state, for example, mood, activity, sleep, stress, medication, alcohol, hallucinations, hash, isolation, exercise, hygiene, paranoia, self-harm, sensitivity, and drugs. A note function allows explaining the assessment scores.	The young adult and the health care provider (HCP) customize the assessment list together. The young adult enters data every day using the app. A reminder is provided given the mental health state has not been reported at 8 pm. Data are stored by the smartphone and transmitted automatically to a study server when internet connectivity is available. At this point, data are visible to the HCPs through the affiliated website.
Visualization	Psychoeducation	Data display of the reported mental health state.	The young adult uses the displayed data to explore relations between symptoms, wellness, and behaviors alone or with the HCP. The HCP has an iPad with wireless internet connection and an external keyboard to access data on home visits.
Early warnings signs	Awareness on changes in health	Display of early signs of exacerbation of illness and suggestions of how to tackle changes to stay well.	The young adult and the HCP identify the relapse signature and drill together and create customized feedback to stay alert to early signs of change in the mental health state.
Triggers and alerts	Notifications of changes in health	Data survey to notify signs of exacerbation of illness and to provide feedback on actions to take to stay well.	The young adult and the HCP set up threshold values together to survey the self-assessment scores, for example, stress level higher than 2 (pretty stressed) on more than 2 consecutive days. When the threshold values are triggered, feedback on actions to take is provided.
Action plan	Strategies to stay in good health	Display of 3 levels of relapse prevention strategies: (1) stay well, (2) what can help, and (3) get help.	The young adult and the HCP customize the action plan together.
Medication overview	Medication management	Reminders and tracking of medication adherence.	The young adult and the HCP produce and update the medication overview together. The young adult reports adherence to medication and changes in medication management. The young adult is indirectly reminded about medication management as part of the self-assessment procedure.
Settings	Customization of resources	Customization of reminders and change of pin code. Access to user guide and a film introducing MindFrame.	The young adult makes changes because of needs and preferences.

### Research Design

The study design was qualitative and constituted the third phase of a participatory design process. The phases of the overall study are available elsewhere [40]. MindFrame was tested as an intervention during the period of January 1 to December 31, 2016. Subsequently, the intervention was evaluated.

### Setting

The setting of the research was OPUS. OPUS is a bio, psycho, and social course of intensive outpatient care in Denmark available to young adults, aged 18 to 36 years, for the first 2 years following diagnosis [7]. The course of care is publicly funded. Effects of the OPUS program have been extensively researched and documented [42-44].

### Intervention

MindFrame was implemented as an add-on tool to regular OPUS care in 1 OPUS clinic in Denmark. The criteria for participation in the intervention were the ability to read Danish and willingness to download and use the smartphone app.

The HCPs provided the young adults the invitations to use MindFrame. The invitation informed that (1) MindFrame had been developed in close collaboration with individuals with schizophrenia and HCPs from OPUS as a collaborative tool to support the everyday management of living with the illness, (2) they could use the app for free and for an unlimited period during the intervention period, (3) it was voluntary to use the app, (4) they could terminate use of the app at all times, (5) early termination of the app would not influence their course of care, and (6) they would be invited to share their views on the usefulness and impact of MindFrame at the end of the intervention period. Invitations to use MindFrame were provided throughout the intervention period. Thus, the length of the intervention and the time when the intervention was applied in the course of care differed from person to person.

When a young adult consented to use MindFrame, they were registered on the MindFrame website, and the smartphone app was downloaded from Google Play or App Store. An install guide was provided for this purpose. A secretary at OPUS made the registrations and handled any install problems. The registration procedure automatically generated an email that was sent to the young adult´s private inbox with a secure log-in code (see Ethics section). The log-in code was used to open the app. Individuals who did not own their own smartphone were offered one to use during the intervention period.

### Training

The HCPs in OPUS were responsible for teaching and guiding the young adults in using and customizing the resources in MindFrame. Therefore, HCPs received training ahead of the intervention period. The first author and a MindFrame software designer conducted the training. The training was group-based and held as a 2-hour hands-on session, where the app and the website were carefully explained and then put into their hands to play around. The HCPs who were unable to partake in the group training were offered a one-on-one session by the first author. After the training session, the HCPs were provided a hard copy of a user guide describing each resource in MindFrame in depth, customization of the MindFrame resources, and how to receive first-level support. The first author was available for questions and supervision throughout the intervention period.

### Evaluation

Following the intervention period, MindFrame was evaluated qualitatively. The evaluation process used for this study was inspired by interpretative hermeneutics. As such, it strove to bring out and manifest what is normally hidden in human experiences and human relations [45]. Data were collected through telephone interviews, which have shown to be productive in qualitative research [46].

All the young adults who had used MindFrame at some point during the intervention period were invited to participate in the evaluation. Thus, the recruitment strategy for the interviews was pragmatic and convenient [30]. The only criterion for participation in the evaluation was willingness to share experiences of MindFrame use by virtue of knowledge. The HCPs in OPUS distributed the invitation, and the first author phoned those consented to be contacted explaining more about the purpose of the evaluation and their rights as study participants. The young adults were encouraged to ask questions and were given time to make a decision on participation. All made their decision immediately and provided written consent. Characteristics of the evaluation sample are outlined in Table 2.

The interviews lasted between 35 and 66 min. They were conducted in Danish and recorded using the TapeACall app from Epic Enterprises. To guide and direct the interviews, a semistructured thematic interview guide [47] regarding personal power, knowledge power, and skills power [48] was used. To encourage the participants to speak freely about their views on how MindFrame contributed in the management of their lives with the illness, interview questions were open-ended. However, at the end of each interview, 15 close-ended questions were posed to work around the concept of empowerment and to prompt more direct answers. Examples of the close-ended questions are “When I use MindFrame I feel more in control” and “When I use MindFrame I become more uncertain of what is right and wrong.” Answering could by default be “yes,” “no,” or “I don´t know,” yet most answered in sentences. Given the questions had not been touched upon in the first part of the interview, the participants were invited to unfold their answers.

### Analysis

An interpretative hermeneutical approach, grounded in the work of the German philosopher Hans-Georg Gadamer, guided the data analysis. A hermeneutic interpretative approach goes beyond mere descriptions to look for meaning embedded in common life practices. These meanings are not always apparent to the participants but can be gleaned from the narratives produced by them [45].

In Gadamer’s perspective, interpretation of meaning is not a stepwise approach. He emphasizes the canon principle that meaning comes from the hermeneutical circle of iteratively moving between part and the whole of the text [49]. Consequently, Gadamer does not provide a method for analyzing text, for example, interview transcripts, audio recordings, observations, and notes [50]. Nevertheless, he states that to obtain understanding, methodological direction through a systematic approach is needed [49].

To provide structure in the process of analysis, 4 tasks grounded in the hermeneutical circle served as a guide. The tasks that were derived from Gadamer’s work and proposed by Fleming et al [50] were as follows: (1) finding fundamental meaning of the text as a whole, (2) exploring parts for meaning, (3) comparing the meaning of the whole with the parts, (4) and identifying passages representative of the interpreted meaning.

Guided by hermeneutical thinking, the analysis began with listening to the tapes multiple times and obtaining a fundamental meaning of the interviews from an empowerment perspective. Then, the fundamental meaning was split into smaller parts that were explored by listening to smaller sections and individual sentences. Using the analytical question “what is said in relation to power,” sections and individual sentences were selected. To obtain meaning from the sections and sentences, they were deconstructed through interpretation, and the interpretations were constantly compared and contrasted with the meaning of the whole. According to Gadamer, there is no understanding without the activity of questioning [49]. Hence, explorative questions were constantly posed to the text in the process of interpretation. To ensure a rigorous analysis, questioning continued until an inner unity, which was free from logical contradictions, had been reached. At this point, categories of synthesized meaning were constructed.

**Table 2:** Characteristics of the evaluation sample.

Characteristics			Statistics
**Gender, n (%)**			
	Male		4 (31)
	Female		9 (69)
Age in years, mean (range)			24.8 (18-36)
**Education in years** **, n (%)**			
	Low: ≤9		4 (31)
	Middle: 10-12		6 (46)
	High: ≥13		3 (23)
**Employment status, n (%)**			
	Employed		7 (54)
	Unemployed		6 (46)
**Living conditions, n (%)**			
	Living alone		8 (61)
	Living with spouse or partner		4 (31)
	Living with family		1 (8)
**Has children, n (%)**			
	No		10 (77)
	Yes		3 (23)
**Support worker, n (%)**			
	No		5 (39)
	**Yes**		8 (61)
		Weekly	4 (50)
		Biweekly	4 (50)
**Medication for mental health issues, n (%)**			
	No		4 (31)
	Yes		9 (69)

### Ethics

In accordance with the Danish law, a formal ethics approval of the study was not required. Authorization by the Danish Data Protection Agency (Datatilsynet) was obtained (2008-58-0028).

The study was consistent with the Declaration of Helsinki [51], meaning that the participants were fully informed about the purpose of the research. The informed consent was obtained verbally and in writing before the enrollment, and information about the right to withdraw from the study was provided. The participants were carefully explained that any withdrawal from the study would not influence their course of care.

MindFrame was established under the standard security approval and procedures of the information and technology department in the specific region in Denmark where it was applied.

## Results

### Use of MindFrame

**Figure 2:**
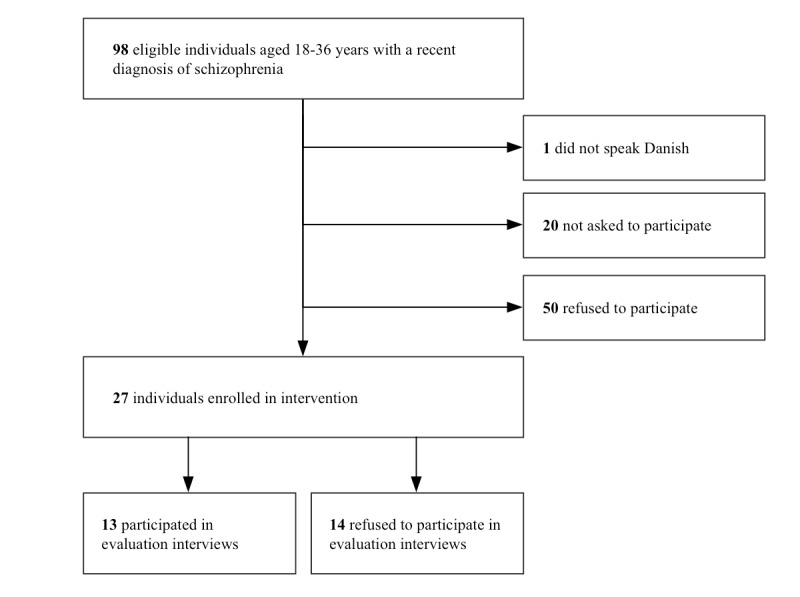
Flowchart of participants in the study.

As evidenced in Figure 2, a total of 98 individuals were eligible to use MindFrame during the intervention period and 27 used it. One of the individuals was excluded from using it as a result of not being able to speak Danish and 50 refused to use it. In 20 cases, individuals were not invited by their HCPs to use MindFrame. On being asked why, the HCPs owed the opt-out decision for exclusions to concerns that these individuals were too ill to use and engage with the app. Out of the 27 young adults who used the app, 13 participated in the evaluation.

The participants in the evaluation described MindFrame as easy and intuitive to use. In accordance with needs and preferences, the period of use of MindFrame differed among the participants. Some participants terminated use within 1 month (n=5), others terminated use within 2 to 3 months (n=4), and others used MindFrame for 6 to 12 months, terminating their use when the intervention period stopped (n=4). Reasons given for self-initiated termination of MindFrame included boredom, lack of motivation and energy, fatigue, and problems quantifying their mental health.

### Perceived Use of MindFrame

On the basis of the participants’ descriptions of use, 2 main and very different categories were generated about the usefulness and impact of MindFrame. When MindFrame was used with HCPs consistently for more than a month, it could provide the participants with the power to keep up their medication, to keep a step ahead of their illness, and to get appropriate help based on their needs. This empowered them to stay on track with the illness, thus in control of it. Furthermore, MindFrame could fuel the fear of restraint and illness exacerbation, thereby disempowering some from feeling certain and secure. This was observed when MindFrame was applied early in the course of care when the participants barely knew their HCP.

Five subcategories led to the 2 main categories. These are outlined in Table 3 and presented in the following section.

### MindFrame Can Provide the Participants With the Power to Keep Up Their Medication

A total of 9 participants received psychotropic drugs for their mental illness during the study. They explained how their memory had been disabled by the illness, yet emphasized how MindFrame had helped them take the medication more regularly. As health tracking covered whether the medication had been taken, not taken, or taken with changes, the self-assessment procedure worked as a daily medication reminder, making it easier to comply with the medication regime. This was a comforting way of staying in control of the medication:

Every day I was reminded to take my medication through the app. That worked really, really well. When I was reminded about it I asked myself, “have you remembered to take your medication today.” If not, I ran out to take it straight away.

Some participants explained how they *forgot* to take the medication deliberately although they knew by heart that they needed it to stay well. One participant who had used MindFrame for 9 months explained how the self-assessment scores had helped her discover that irregular consumption of medication impacted her mental health state. Insight into this pattern of behavior helped her to make the decision to resume her medication regime:

Sometimes the scores made me realize that I needed to take my medication. It is easier to make decisions on [...] resuming taking the pills when I can see that my symptoms are progressing when I don´t take them.

**Table 3:** The hermeneutical-inspired process of analysis governing the findings.

Words on tapes (quotations)	Immediate answers: what is said in relation to power?	Decontextualization through interpretation: empowering aspects of MindFrame	Categories	Result and major categories
*When I first started in OPUS I had problems with compliance. MindFrame helped me to remember to take my pills.*	*Helped me to remember to take my pills.*	Power to maintain medication	MindFrame can provide the participants with the power to keep up their medication	MindFrame can empower the participants to stay on track with the illness
*Triggers and alerts made me conscious about signs I had to pay attention to, and act upon to stay well [...].* *I believe that has helped me to stay on track.*	*Made me conscious; pay attention; act [...] to stay well; stay on track.*	Power to act timely to stay on the track of health	MindFrame can provide the participants with the power to keep a step ahead of their illness	MindFrame can empower the participants to stay on track with the illness
*MindFrame helped me to get anti-depressants pretty fast, when I needed it. I don´t believe that would have happened if my psychiatrist had not had the chance to look at my scores.*	*Helped me [...] fast; when I needed it.*	Ability for health care providers (HCPs) to be more responsive to needs	MindFrame can assist the participants with the power to get appropriate help based on their needs	MindFrame can empower the participants to stay on track with the illness
*If my scores were really bad, then, could they [HCPs] use my scores to put me under restraint? I was really uncertain of that in the beginning.*	*Could they put me under restraint?; really uncertain.*	Lack of power to feel secure	MindFrame can increase participant fears and worries of restraint	MindFrame can also fuel the fear of restraint and illness exacerbation, thereby disempowering some participants from feeling certain and secure
*Triggers and alerts gave attention to any early signs of change. What is this? Am I getting worse? That made me worried at some point.*	*What is this?; Am I getting worse?; made me worried.*	Lack of power to feel certain	MindFrame can increase uncertainties in the participants about their mental health state	MindFrame can also fuel the fear of restraint and illness exacerbation, thereby disempowering some participants from feeling certain and secure

Thus, MindFrame seemed to provide the participants receiving psychotropic drugs the power to keep up with their medication so as to stay well. This was the case when the self-assessment procedure was used passively as a reminder to take medication or when the self-assessment scores were used actively to make the decision that medication should be resumed to stay on track.

### MindFrame Can Provide the Participants With the Power to Keep a Step Ahead of Their Illness

The participants stressed how they had to react quickly to early signs of exacerbation of illness to prevent symptoms from progressing into full psychosis. The participants who had set up threshold values for triggers emphasized how MindFrame was a powerful resource to this end. They explained how their scores had prompted a trigger, which alerted them to be aware of the early signs of change, causing them to act upon these signs to stay in good health:

The trigger and alert function was really smart. It showed when things changed, and made one aware to do something in order to stay well.

Awareness was brought to mind automatically through the visualization feature in MindFrame even when threshold values had not been set. Most of the participants made self-assessments on a daily basis for a period of time during the intervention period and emphasized how the display of their scores helped them to see *when* they should behave differently to stay well. This encouraged the belief that the illness would remain within their control:

It is so comforting that I know that the system shows me if the illness is getting worse. Then I know when I should act to prevent it from getting out of control.

To stay in control of the illness, it was not enough to know *when* action should be taken. Knowing *which* action should be taken and *how* to stay on track were equally important. A few of the participants had customized their action plan with their HCPs, and they explained how the plan of action had provided them with strategies to stay well, saying, “The action plan tells me what to do to stay well.” Other few participants had used the action plan without customization, which some found useful.

Thus, MindFrame seemed to provide the participants with the power to keep a step ahead of their illness rather than at the rare end of it by making them aware of when to act and how. This was the case when self-assessments had been conducted for more than a month and especially the case when the self-assessments were used with triggers and a customized action plan.

### MindFrame Can Assist the Participants With the Power to Get Appropriate Help Based on Their Needs

All the participants described how cognitive difficulties challenged them when trying to remember how their health had been over time. In this respect, they stressed how MindFrame had empowered their memory to keep track of their state and progress:

It [MindFrame] helps me a great deal when remembering how I was last week or a couple of weeks ago. I cannot find back to how things were, MindFrame has helped me to keep track of this: how it was.

The ability to keep track of their mental health state and progress was strongly emphasized by the participants, as it provided a solid basis for discussing their health and needs of care with the HCPs. To this end, most underlined how mental health tracking assisted their HCPs to ask more direct questions about the fluctuations in their mental health and relations between the mental health state and their behaviors and actions:

It has been easier for [name of HCP] to ask questions since she could see my scores: “I can see that you have had a bad day what happened that day?” She knew how my week had been and could ask more direct questions.

Ultimately, this contextualized dialogue enabled the HCPs to be more responsive to the needs of the participants, which empowered them to receive the help they needed when they needed it:

I was in a period where my thoughts were getting darker and darker and [name of HCP] said to me: “I can see from your scores that your mood and sleep is not good at the moment. I don´t think your antidepressants help you enough.” She was right. Then the dose was increased, and after some time I started to get better.

As such, MindFrame seemed to provide the participants with the power to get appropriate help based on their needs. This was the case when they reported their mental health state and the scores were used by the HCPs as a basis for assessing and adjusting care to their needs.

Several of the participants emphasized how they wanted their HCPs to take even more advantage of using their scores in their course of care. They explained how looking at the scores with the HCPs and getting expert help to add meaning to the score enabled them to better understand the causations of fluctuations in the mental health state and allowed the effectiveness of behavior change to be evaluated. They believed that learning generated from their own data could equip them to more confidently and independently navigate the everyday management of the illness in the long run.

### MindFrame Can Increase Participant Fears and Worries of Restraint

As evidenced in the previous section, it seems that MindFrame could provide the participants with the power to stay on track of their illness. However, it also seemed that MindFrame could increase fears and worries in some of the participants, thereby disempowering them from feeling certain and secure. This was observed in 3 participants who had just been enrolled in OPUS and had only known their HCP for a short period. Shared for these participants were concerns of using MindFrame even before beginning its use. They stressed how they were worried that their HCPs could survey their mental health state on a day-to-day basis or keep them under *surveillance* in the time between consultations. They feared that surveillance could lead to situations where they were unwillingly put under restraint and committed to hospital:

My biggest concern about starting using MindFrame was that my nurse would observe my condition every day. Then, would there be consequences? Could she use my scores to admit me to the hospital?

The fear of surveillance seems to fade with use*.* Two of the 3 participants stressed how concerns and fears had become less dominant over time as they had become more familiar with MindFrame and certain about the fact that their HCPs were only interested in their scores to provide the best possible care:

At first I was a bit worried that [name of HCP] could see all my scores, but when I found out that she was only interested in my scores to help me my worries disappeared.

In 1 of the 3 participants, fears and concerns of restraint remained. Consequently, this participant did not report his true state of mind on his bad days. Rather, he touched up the scores making his mental health seem better than it was. This participant stopped using MindFrame within 1 month. The rest of the sample did not address fears and worries of restraint in relation to their mental health state being observed by their HCPs. Rather, they talked about surveillance of their mental health as a way of careful watching, helping them to get timely and appropriate help based on their needs.

Thus, for some participants, MindFrame seemed to increase fears and worries of restraint, which prevented them from feeling confident and safe. Worries and fears seemed to fade with use of the app but remained with 1 participant who embellished his data to stay in control.

### MindFrame Can Increase Uncertainties in the Participants About Their Mental Health State

MindFrame seemed to provide the participants with the power to keep a step ahead of their illness, thus staying on track. Being a step ahead of the illness, however, was not always perceived positively. Two participants who had conducted self-assessments continuously for several months addressed this. Both participants felt that the notifications felt comforting and allowed them to act timely; however, occasionally it was stressful to be alerted about *all* the changes in their mental health state, as it left them wondering if their condition was worsening:

Being notified of all the changes sometimes made me anxious. It made me wonder if the illness was maybe about to get out of control.

The 2 participants explained that doubt and hesitation about their mental health state was something they dealt with on a daily basis, thus it was not something new. However, they stressed how the notifications in some ways increased their uncertainty. They experienced this when there were incongruences between their perception of their mental health state and the state communicated by MindFrame. When their personal interpretation of the information gained from their senses did not match the notifications from MindFrame, they were left in doubt of what to think and whether or not to act:

When the notifications tell me to take care and I feel fine, it makes me question myself even more. Is it ok now, or should I do something?

Thus, for some participants, MindFrame seemed to increase uncertainties regarding their mental health state and thereby disempowered them from feeling self-confident and on track with their illness. It was only observed in 2 participants, and they stressed that their uncertainty often disappeared when the notifications were shared and discussed with their HCPs.

## Discussion

### Principal Findings

This study explored how young adults recently diagnosed with schizophrenia used and perceived the smartphone app MindFrame as a tool to foster power in the everyday management of living with their illness. Findings from the interviews showed that when MindFrame was used continuously for more than a month and with the HCPs, the participants were provided with the power to keep up their medication, to keep a step ahead of their illness, and to get appropriate help based on their needs. This empowered them to stay on track with the illness, thus in control of it.

The findings showed that prolonged and continuous self-assessments were main components responsible for the efficiency of MindFrame. When data were collected consistently over a period of time, a picture of the mental health state of the participant was generated, and this picture worked as a tool to inform decisions about medication and as a tool to alert timely actions to stay in good health. In addition, prolonged and continuous self-assessments worked as a tool to inform the HCPs about the mental health state of the participant, which enabled them to deliver timely care more responsive to their needs. The findings highlight that as a tool to foster power in the everyday management of living with schizophrenia, MindFrame is mostly viable in young adults with schizophrenia who are willing, able, and capable of assessing their health over the course of time. Tenacious use of smartphone apps in the care for persons with schizophrenia may be difficult to obtain [52-55], which was also evident in our study where 5 out of 13 participants terminated use of MindFrame within the first month. This was true, although the resources in MindFrame were closely aligned with the needs and preferences of the intended user group, which is suggested to foster engagement [53,56-58]. This shows that MindFrame—despite being codesigned—was neither applicable nor appealing to all. The fact that only approximately 35% (27/77) of those invited to use the app accepted to use it further underlines this and indicates that MindFrame may not generalize to the broader population of young adults recently diagnosed with schizophrenia. Further research is needed to establish this.

The findings showed that collaborative use of MindFrame was another main component of its efficacy. When the self-assessment scores of the participants were shared with their HCPs, the HCPs were enabled to deliver care more responsive to their needs, which empowered them to stay on track. The participants stressed how they wanted their HCPs to take even more advantage of using their scores in their course of care. They believed that learning generated from their own data could equip them with the knowledge and skills to more confidently and independently navigate the everyday management of the illness in the long run. In line with previous research, the findings indicate that HCPs are responsive to integrating smartphone technology into young adult schizophrenia care [25], yet, that HCPs uptake could be better [59]. Successful implementation and dissemination of smartphone apps as part of schizophrenia care for young adult population will rely on provider uptake as well as client use [25]. Future research will need to address how to increase provider uptake and evaluate the impact of provider engagement on the ability to navigate the everyday management both in the short and long run.

As evidenced, the findings suggest that MindFrame can be used as a tool to foster power in the everyday management of living with schizophrenia. However, we identified 2 key aspects of use to take into account.

First, we identified that MindFrame could increase fears and worries of restraint, thereby disempowering some participants from feeling certain and secure. The fears and worries were related to data sharing when participants did not know their HCP very well. Ben-Zeev et al [14] investigated passive monitoring through sensors in a smartphone app. Using a sample of 11 inpatients and 9 outpatients with schizophrenia, for 1- or 2-week periods, respectively, they observed that approximately 20% of the sample felt upset by monitoring. This substantiates that worries related to health monitoring are rather common in individuals with schizophrenia even when data are generated automatically. We found that the feeling of uncertainty blurred when the participants got more familiar with the monitoring aspect of the app and their HCPs. This suggests that certainty may develop with use over time. However, we found that 1 participant embellished his data to stay in control, which accentuates that this might not always be the case. This advocates that health monitoring may have its limitations and highlights the paramount importance of carefully assessing the most appropriate time in the course of care to introduce and use an app for empowering purposes. Future research will need to look closer into the characteristics of those feeling upset from monitoring to fully understand its limitations.

Second, we identified that MindFrame could increase uncertainties about participants’ own mental health state, thereby disempowering some from feeling certain and secure. The uncertainty was related to notifications of exacerbations of illness and arose when the app indicated worsening, but the participant was fine. The findings show that being notified may lead to an emotional response of disturbance when the notification does not correspond to the participant´s sense of health. The same was identified in individuals with severe and very severe chronic obstructive pulmonary disease. Hunicke et al found that disturbance arose when individuals felt better or worse than what the technology indicated [60]. The former is in line with our findings and highlights how monitoring may increase uncertainty even in individuals who have been living and managing their illness for a long time. The finding highlights the paramount importance of using an app as part of a collaborative partnership with the HCP to increase certainty. HCPs have clinical knowledge and insights of importance that would help young adults diagnosed with schizophrenia set up the right threshold values to notify changes in their mental health state and to adjust the values as the illness stabilizes or exacerbates. Given the young adult is left alone to do this, it is likely that the amount of false-negative or false-positive notifications may increase. Ben-Zeev et al stress that in the future, evidence-based mHealth apps will be downloaded directly onto the smartphone and used by individuals with little or no contact to mental health care facilities [28]. Our findings suggest that in the case of young adults recently diagnosed with schizophrenia, this may leave some worried and uncertain.

Schermer has sketched 2 possible future scenarios of the use of smartphone technologies in mental health care. One scenario is the *Big Brother scenario*, where monitoring technology will reproduce the old paternalistic paradigm of patient-HCP interaction in which compliance and monitoring are the aims. The other scenario is that it will create a new situation that centers on shared decision making and self-management, adding to the autonomy of the service user [61]. Our findings suggest for the latter scenario to be feasible.

### Limitations

A number of key limitations must be acknowledged. The recruitment strategy restricted 20 individuals from choosing for themselves whether or not to engage in the intervention. Opt-out decisions where HCPs set up their own criteria for excluding individuals with mental health issues from participation in interventions appear rather common [62-64]. In our study, this may have contributed to an evaluation sample nonrepresentative of the population and the impression that MindFrame may not generalize to the broader population of young adults with a recent diagnosis of schizophrenia.

The evaluation sample was small, and most of the participants had positive attitudes toward MindFrame. The poor retention of study participants may have overvalued the positive effects of the technology. A replication of the study with a larger sample size and maximum variation sampling in the interviews could help clarify this. Contrary to convenience sampling, maximum variation sampling and extreme case sampling allow the researcher to purposefully select participants to learn from the most extreme and unusual cases [30].

The evaluation sample was one of convenience and consisted of 9 women and 4 men. Research has established that first episode schizophrenia incident rates are approximately 2 times higher in men than in women [65,66]. This suggests that our findings are gender biased and potentially in favor of women. A replicative study with a sample more representative of the population would be interesting to see whether these study findings are gender consistent. This might not be the case, as previous research has provided findings that male gender is a specific predictor of nonadherence to mHealth interventions [53].

The sample was interviewed post intervention. For the participants who had terminated using MindFrame after a short period, the evaluation was conducted several months after they had stopped using it. Given the cognitive deficits addressed in the analysis and broadly in the scientific literature [67,68], it is possible that our study design has contributed to recall bias, which may have prevented some complexities from unfolding. The research process, however, does not indicate this. When interview questions were posed, the participants easily shared their views and experiences.

### Conclusions

Our findings demonstrate that young adults recently diagnosed with schizophrenia are amenable of using a smartphone app as part of their everyday life to monitor their health, to manage medication, and to stay alert of early signs of exacerbation of illness. Given the app is used consistently for more than a month and in close collaboration with HCPs, it may empower them to keep the illness within their control.

The findings encourage the application of smartphone-based care to aid this population to better help themselves in the time following the diagnosis. The disempowering aspect of MindFrame accentuates that a smartphone app should be used in a reflected manner at the right time in the course of care and with the right amount of support. Further research is required to understand the best uptake and limitations of smartphone-based young adult schizophrenia care.

## Abbreviations

HCPs: health care providers
mHealth: mobile health
